# Evaluation of the Inverted Classroom Approach in a Case-Study Course on Antithrombotic Drug Use in a PharmD Curriculum: French Monocentric Randomized Study

**DOI:** 10.2196/67419

**Published:** 2025-04-10

**Authors:** Georges Jourdi, Mayssa Selmi, Pascale Gaussem, Jennifer Truchot, Isabelle Margaill, Virginie Siguret

**Affiliations:** 1 Optimisation thérapeutique en neuropharmacologie, INSERM U1144, Université Paris Cité, Paris, F75006, France; 2 Service d’Hématologie Biologique, Hôpital Lariboisière, AP-HP.Nord, Paris, France; 3 INSERM UMR-S970, Paris Cardiovascular Research Center, Université Paris Cité, Paris, F75015, France; 4 Service d’Hématologie Biologique, Hôpital Européen Georges Pompidou, AP-HP.Centre, Paris, F75015, France; 5 Emergency Department, Hôpital Cochin, AP-HP.Centre, Université Paris Cité, Paris, France; 6 iLumens, Université Paris Cité, Paris, France; 7 US UPPERS, Faculté de Santé, Unité de Service Pour la Pédagogie, l'Enseignement et la Recherche, Université Paris Cité, Paris, France

**Keywords:** antithrombotic drugs, case-study course, inverted classroom, pharmacy students, traditional educational approach, medical education

## Abstract

**Background:**

Appropriate antithrombotic drug use is crucial knowledge for pharmacy students.

**Objective:**

We sought to compare the inverted classroom (IC) approach to a traditional question-and-answer educational approach with the aim of enhancing pharmacy students’ engagement with a case-study course on antithrombotic drug use.

**Methods:**

Third-year PharmD (Doctor of Pharmacy) students from Paris Cité University were randomly assigned to control (n=171) and IC (n=175) groups. The latter were instructed to read and prepare the preprovided course material 1 week before the in-class session to assume the instructor role on the target day, whereas students of the control group attended a traditional case-study course carried out by the same instructor. All students completed pre- and posttest multiple-choice questions surveys assessing their knowledge levels as well as stress, empathy, and satisfaction questionnaires.

**Results:**

A significantly higher participation rate was observed in the control group (93/171, 54%) compared to the IC group (65/175, 37%; *P*=.002). Women (110/213, 52%) participated more than men (48/133, 36%; *P*=.002) whatever the group was. Students’ knowledge scores from both groups had similar results with no difference neither in the prescore (1.17, SD 0.66 and 1.24, SD 0.72 of 5, respectively) nor in the short-term knowledge retention (2.45, SD 0.61 and 2.35, SD 0.73, respectively). The IC approach did not increase student stress or enhance their empathy for the instructor. It increased the preclass workload (*P*=.02) and was not well received among students.

**Conclusions:**

This study showed that the traditional educational approach remains an efficient method for case-study courses in the early stages (ie, third-year) of the 6-year PharmD curriculum, yet dynamic methods improving the active role of students in the learning process are still needed.

## Introduction

The French Regional Centers of Pharmacovigilance recently revealed that 8.5% of hospital admissions of 141 short-stay specialist medical wards randomly selected in 69 public hospitals, were related to adverse drug reactions [1]. Antithrombotic drugs were involved in 11.6% of the cases, placing them in second place right behind antineoplastic drugs. Some of the adverse drug reactions (mainly bleeding) were considered to be preventable because the drugs had not been used per the summary of product characteristics or guidelines [2]. In the French health care system, antithrombotics are mainly available in pharmacies, and for some also on websites but under the responsibility of a pharmacist. Pharmacists, particularly community-based pharmacists, are easily accessible health care professionals [3]. They are experts in drug therapy use, assessing each patient through observation, dialogue, and consideration of clinical indicators. They are involved in monitoring the patient’s compliance with treatment as well as their response to drug therapy through regular follow-up. This allows for the early detection of adverse effects or drug misuse. Therefore, pharmacist intervention should have a positive impact on the management of patients on antithrombotic therapy. All the above reasons makes the optimal use of antithrombotic drugs of utmost importance to learn for pharmacy students to prevent iatrogenesis.

In France, the PharmD (Doctor of Pharmacy) curriculum consists of a 6-year course. Three teaching models are used mainly: the lecture-based classroom, the case-study class, and the practical session. Case-study class is a hands-on approach to learning that involves presenting realistic scenarios and helps students to apply theoretical knowledge in clinic-like settings and attain a high-order cognitive level per Bloom’s taxonomy [4]. The instructor asks students to participate in the case study analysis and discussion. This method favors the development of a deep understanding of the subject and avoids passive note-taking in students. However, this objective is not necessarily always reached. Since its introduction in 2000, the inverted classroom (IC) approach, switching away from the traditional educational approach for the lecture-based classroom, literally inverts the focus per Bloom’s taxonomy: the bottom parts of the taxonomy (ie, understand and memorize basic concepts) are reserved for student self-instruction through readings, short recorded video, audio lectures, etc, while the class time is focused on the upper parts of the taxonomy (ie, analyze, justify a stand, and create original work). The IC approach has been increasingly studied in health professions students’ education including pharmacy school [5-17]. These studies reported a positive impact of this approach on students’ knowledge and skills in most of the cases in comparison to lecture-based courses [8-11,13,14,18-24]. It has been introduced into various courses including pharmacotherapy, pharmacokinetics, pharmaceutical calculations, pharmacy practice, and others [7,12,25-32]. That said, the added value of the IC approach has rarely, if at all, been tested for case-study courses in pharmacy education. Hence, we sought to conduct a monocentric study investigating the added value of an IC approach in a case-study course during the PharmD curriculum at Paris Cité University. In this IC approach, students received the course material before the in-class session and were asked to prepare it and assume the role of the instructor on the target day. We aimed to assess knowledge acquisition, preclass workload and students’ self-assessment of their stress, empathy, and global satisfaction. It was hypothesized that the IC approach would lead to improved outcomes compared to the traditional question-and-answer educational approach.

## Methods

### Ethical Considerations

This study was approved by the Institutional Review Board and Ethics Committee of Paris Cité University (00012023‐20) and all procedures were performed per the Helsinki Declaration. Students were completely free to participate or not in this study. They were informed that neither participation nor nonparticipation in this study would influence their passing of this course or their grades. The participants were also informed about the option to withdraw from this study at any time. Informed consent was obtained from all the students who participated in this study. They were not paid for their participation. Collected data were anonymously analyzed.

### Participants

**Figure 1.**
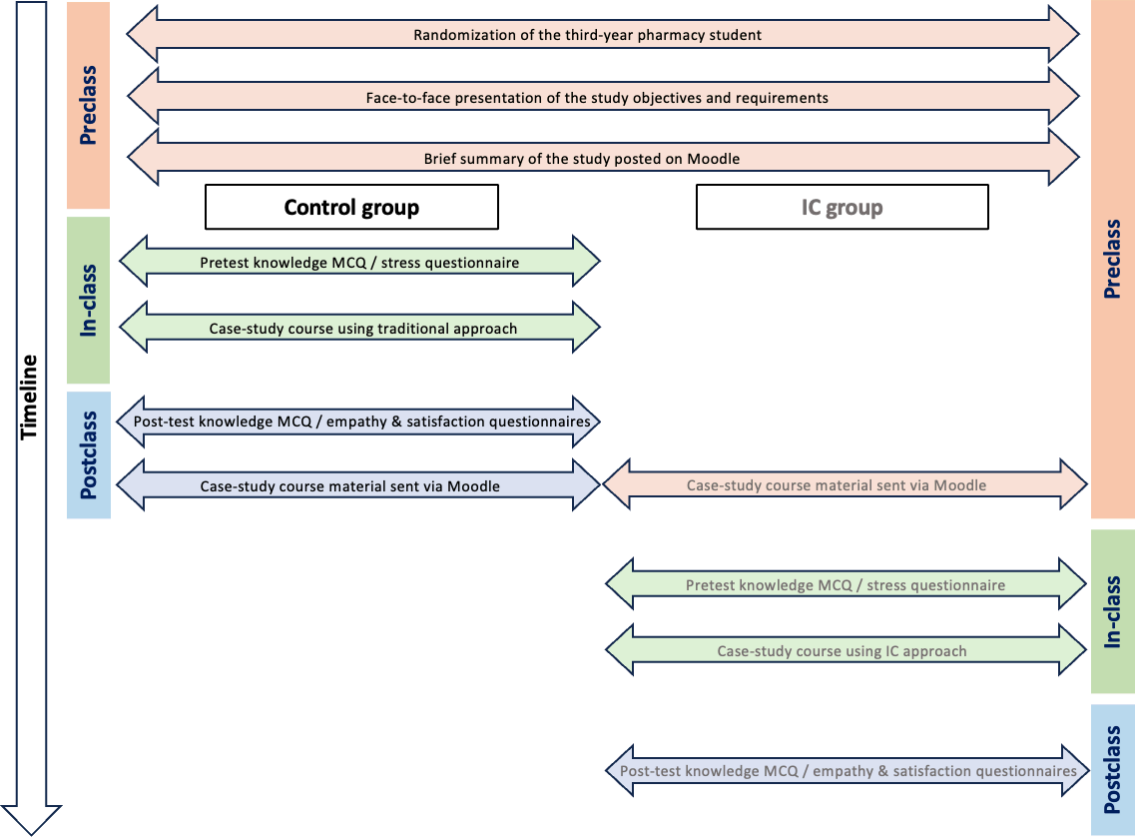
Overview of this study’s design. IC: inverted classroom; MCQ: multiple-choice question.

We conducted this study at the faculty of Pharmacy of Paris Cité University in March 2023 (2022/2023 academic year). The participants were in the spring semester of the third year of the PharmD curriculum. At the beginning of the semester, students had basic courses on hemostasis (physiology and pathology) and thrombosis. During the month preceding the case-study course, they also had 3 lecture-based courses for a total of 4 hours on antithrombotic drugs. The recordings of the lectures were then available on a teaching platform (“Moodle”). The 90-minute case-study course on antithrombotic drug use entailed 16 groups consisting of about 20 to 22 students each, which were randomly assigned to the groups by the office of student affairs of the faculty of Pharmacy without any influence from the instructor. Therefore, a randomized assignment can be assumed. Students were highly required to respect their group assignment. Each time, 2 groups assisted simultaneously in one course, thus the course was repeated 8 times by the same instructor. Participants were assigned to 2 major groups: the control group and the IC group (Figure 1). Attendance was not mandatory.

### Study Design and Procedure

Three weeks before the case-study course, the instructor informed all the students about the concept of the IC approach and this study’s process in addition to the importance of their engagement in the learning process. A summary of the process was also posted on Moodle. Students were informed that half of them would be assigned to the IC group where they would receive the course material for preparation 1 week before the in-class session to take the role of the instructor on the target day. The course material was delivered as a PDF document of a PowerPoint (Microsoft Corp) presentation (encompassing 63 slides) file via Moodle. The same course material was used in both approaches. The courses took place on Monday and Tuesday. One week separated the courses of the control from the IC groups.

Six cases concerning various clinically relevant scenarios were included in this course in addition to some incorporated slides to review and emphasize selected foundational concepts. They were centered on the most relevant aspects of antithrombotic drug use in different clinical settings: (1) treatment of pulmonary embolism associated with proximal deep vein thrombosis in a posttrauma patient aged 63 years; (2) prevention of thrombotic events postelective knee replacement surgery in patient aged 78 years; (3) treatment of pulmonary embolism that occurred under combined pill contraception in a woman aged 20 years; (4) prevention of thrombotic events in an acutely ill medical patient aged 80 years; (5) prevention of stroke and systemic embolism in a patient aged 82 years with atrial fibrillation and renal insufficiency; and (6) antithrombotic treatment of myocardial infarction in a patient aged 54 years. Each case was accompanied by a set of standardized questions about antithrombotic treatment decisions and adequate monitoring. Students in the control group attended a case-study course carried out by the instructor with a traditional question-and-answer approach, whereas students in the IC group took on the role of the instructor and were able to recall basic concepts or add details to the course material freely. Two to three students were randomly asked to present and discuss 1 of the 6 cases on the target day. By doing so, students were able to draw on each others’ knowledge and understanding. The instructor added details, provided guidance, clarity and feedback whenever required during the progress of the students’ presentation and summarized the main features at the end of each case. The classes in the control and IC groups were conducted by the same experienced instructor who is familiar with the content and organization of the case-study course to guarantee the consistency of the teaching content and objectives in the 2 educational approaches. This study’s design and progress are illustrated in Figure 1.

### Data Collection

On the target day, students in both control and IC groups were asked to complete a pretest (ie, at the beginning) and a posttest (at the end) survey (Multimedia Appendix 1): it consisted of the same 5 multiple-choice questions (MCQs) to be completed within 5 minutes, then collected in an identified way (ie, including the student name, and the date and the hour of the questionnaire completion) to pair pre- and posttest scripts. Questionnaires were anonymized by a secretary and corrected afterward by an independent assistant instructor. Another 5-minute survey assessing the stress in the week preceding the in-class course (thus assessing how they were affected from the moment they knew which group they belonged to till the target day) was also completed by all the students at the beginning of the course. At the end of the course, students were asked to complete 2 additional surveys, 1 assessing their empathy for the instructor and the other their global satisfaction. The first consisted of rating 3 items by a 7-point Likert scale (1=strongly disagree, 2=disagree, 3=somewhat disagree, 4=neutral, 5=somewhat agree, 6=agree, and 7=strongly agree). The second included 3 questions regarding the preclass workload associated with the educational approach and the related students’ perception, and 6 others linked to the students’ satisfaction with the course objectives, course material, in-class progress, and educational approach. Surveys were built based on previously validated assessment tools [33-35]. Stress, empathy, and satisfaction surveys (MultimediaAppendices 2^4^) were filled out anonymously. Students were not allowed to keep a copy of the different surveys nor to take a photo of these documents.

### Statistical Analysis

The distribution of the data was evaluated using the Shapiro-Wilk test. The percentage of participation was compared between groups and sexes using a 2-way ANOVA followed by the 2-stage setup method of Benjamini, Krieger, and Yekutieli [36] for multiple comparisons. The results of the pre- and posttest MCQs were compared for statistically significant differences using the nonparametric Wilcoxon matched-pairs signed rank test. The preclass workload was compared between the 2 groups using the Mann Whitney test. The data relative to the empathy and stress self-assessment were compared between both groups using chi-square test whereas those relative to satisfaction were analyzed using the Fisher exact test. Error probability with a *P* value less than .05 was considered significant. Statistical analysis and graphical representation were performed using GraphPad Prism (version 10.0.2, GraphPad Software, Inc).

## Results

### Participants Characteristics

In this study, 346 third-year adult students (women: n=213, 62%; men: n=133, 38%) were randomized. As attendance is not mandatory, only 46% (n=158) attended the in-class session. All of them took part in this study. Ninety-three (women: n=70, 75%; men: n=23, 25%) were in the control group whereas 65 (women: n=40, 62% women; men: n=25, 38%) were in the IC group (Table 1). A significantly higher participation rate was observed in the control group (93/171, 54%) compared to the IC group (65/175, 37%; *P*=.002). Women (110/213, 52%) participated more than men (48/133, 36%; *P*=.002) in the case-study course no matter the allocation group. No other demographic information regarding the students was collected.

**Table 1.** Characteristics of the participants. Absolute numbers with the percentages concerning the corresponding randomized participants are reported. Women participated more than men in the case-study course whatever the group was (*P*=.002) and a significantly higher participation was observed in the control group compared to the IC^a^ group (*P*=.002).

	Sex	Control group	IC group	Total
Randomized participants				
	Men	55	78	133
	Women	116	97	213
	Total	171	175	346
Effective participants, n (%)				
	Men	23 (42)	25 (32)	48 (36)
	Women	70 (60)	40 (41)	110 (52)
	Total	93 (54)	65 (37)	158 (46)

a IC: inverted classroom.

### Pre- and Postclass Knowledge Survey

**Figure 2.**
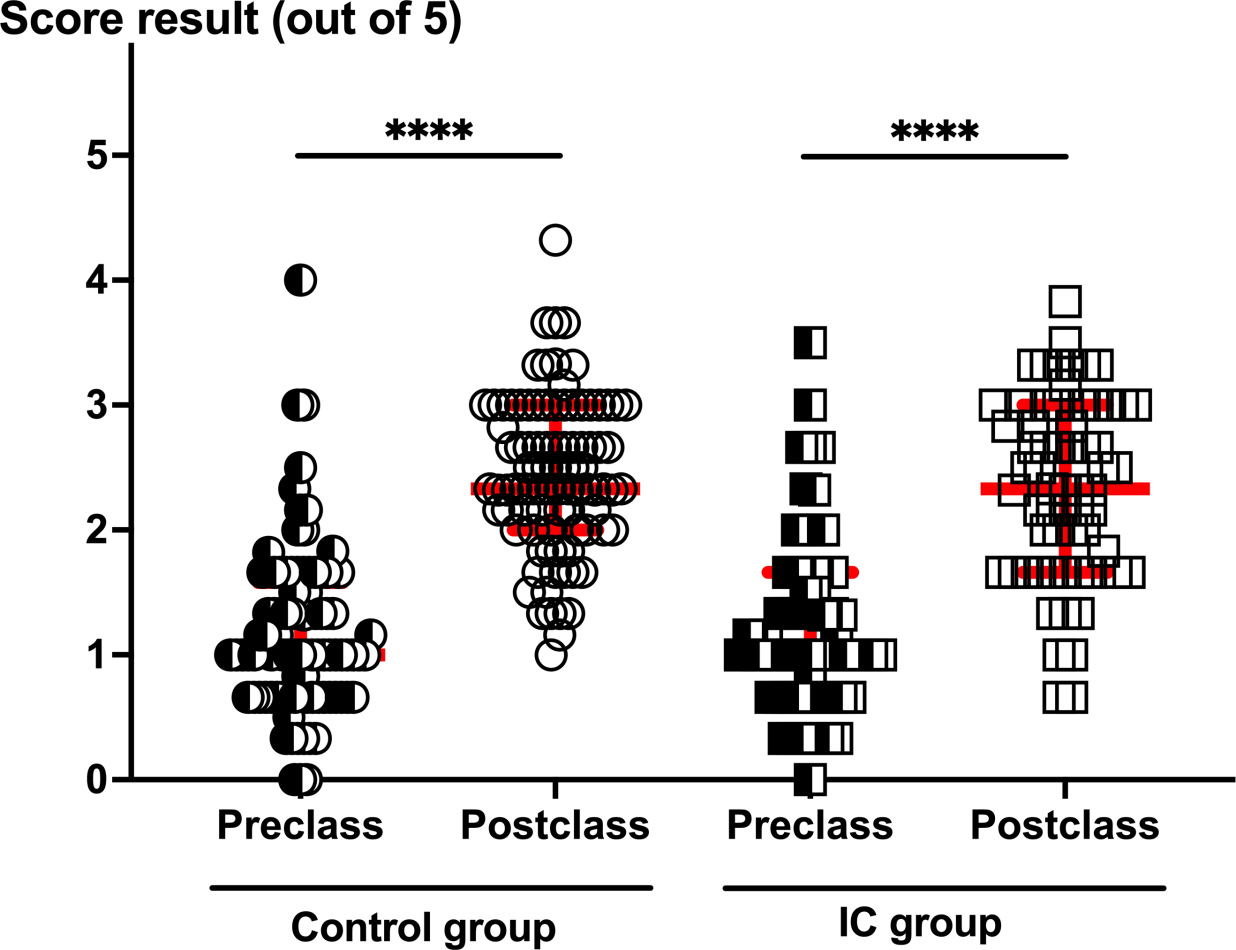
Pre- and postclass knowledge assessment survey per group. Five multiple-choice questions were completed by the control (circles, n=93) and IC (squares, n=65) groups before (semiclosed symbols) and after (open symbols) the completion of the case-study course. Red bars reflect median values with IQRs. Students’ scores significantly increased (*P*<.001) at the end of the course in both groups. No difference in the scores was observed neither at the beginning nor at the end of the course between the 2 groups. IC: inverted classroom.

On the target day, the in-class session started for both groups with a 5 MCQ survey for 5 minutes to assess the students’ readiness to discuss cases and stimulate the recall of knowledge learned before the case-study course. Questions were on the “take-home messages” related to antithrombotic drug use in real-life clinical settings. These MCQs also help students to identify their possible misconceptions at the beginning of the course. The mean and SD of the prescore (out of 5) did not differ between the control group (1.17, SD 0.66) and the IC group (1.24, SD 0.72; Figure 2). To evaluate the students’ short-term knowledge retention, the same survey was completed immediately after the class. The mean score improved from the prescore (*P*<.001), in both the control group 2.45 (SD 0.61) and the IC group 2.35 (SD 0.73; Figure 2). Knowledge improvement was comparable in students from both groups.

### Stress Self-Assessment

**Figure 3.**
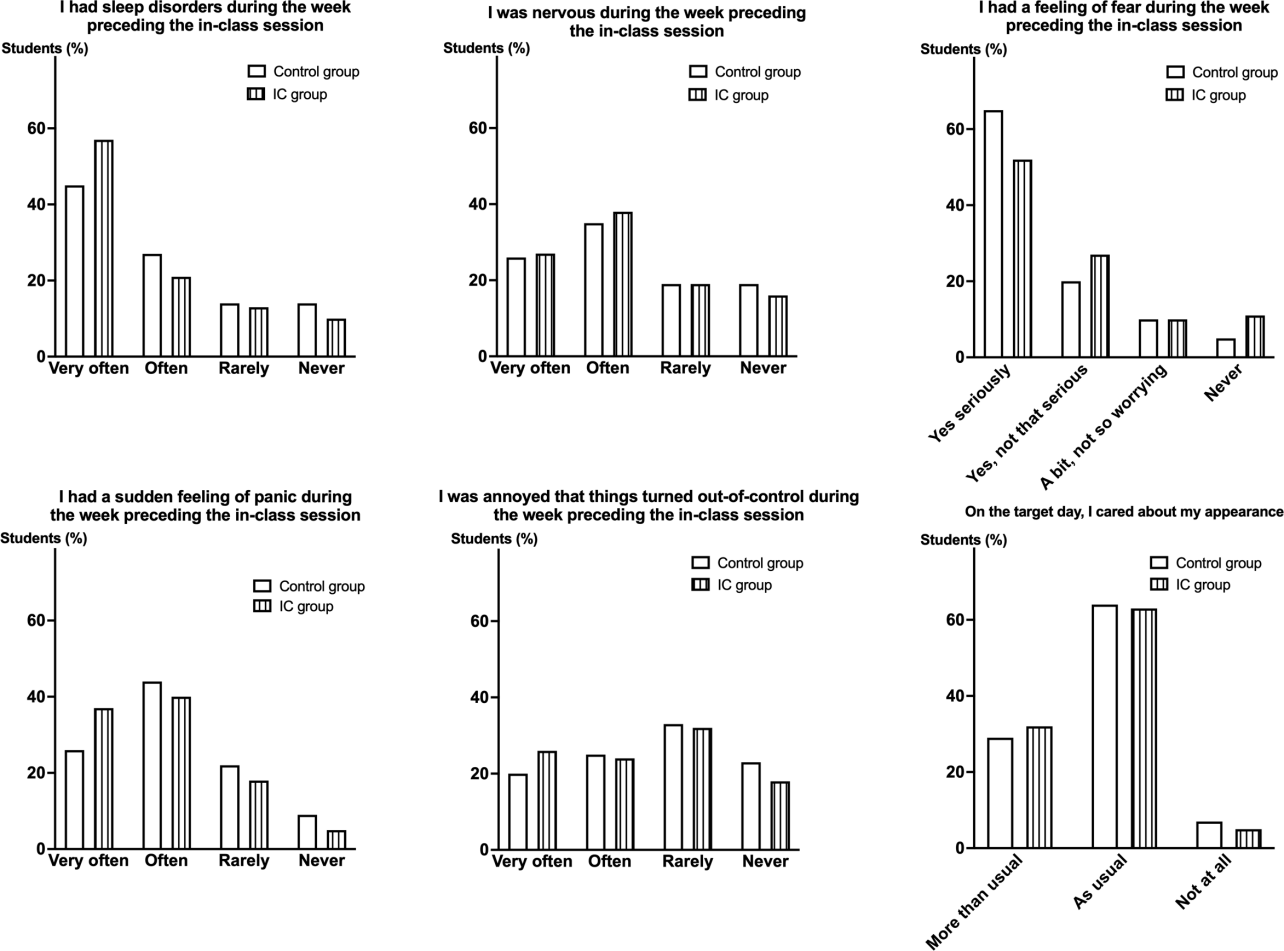
Stress self-assessment questionnaire. A 6-question stress self-assessment survey was completed by both the control (n=93) and IC (n=65) groups at the beginning of the case-study course. No significant difference for any of the 6 questions was observed between the 2 groups. IC: inverted classroom.

Apart from the preclass knowledge assessment survey, students in both groups completed a stress survey at the beginning of the class during 5 minutes. Questions related to sleep disorders, nervousness, fear, panic, and annoyance during the week preceding the in-class course were completed (Figure 3). Although approximately 50% of the students reported sleep disorders and a feeling of fear during the week preceding the in-class session, no significant difference was observed between the 2 groups for any of the above-mentioned parameters. Likewise, students care for their appearance on the target day did not differ between the control and the IC groups.

### Preclass Workload

**Figure 4.**
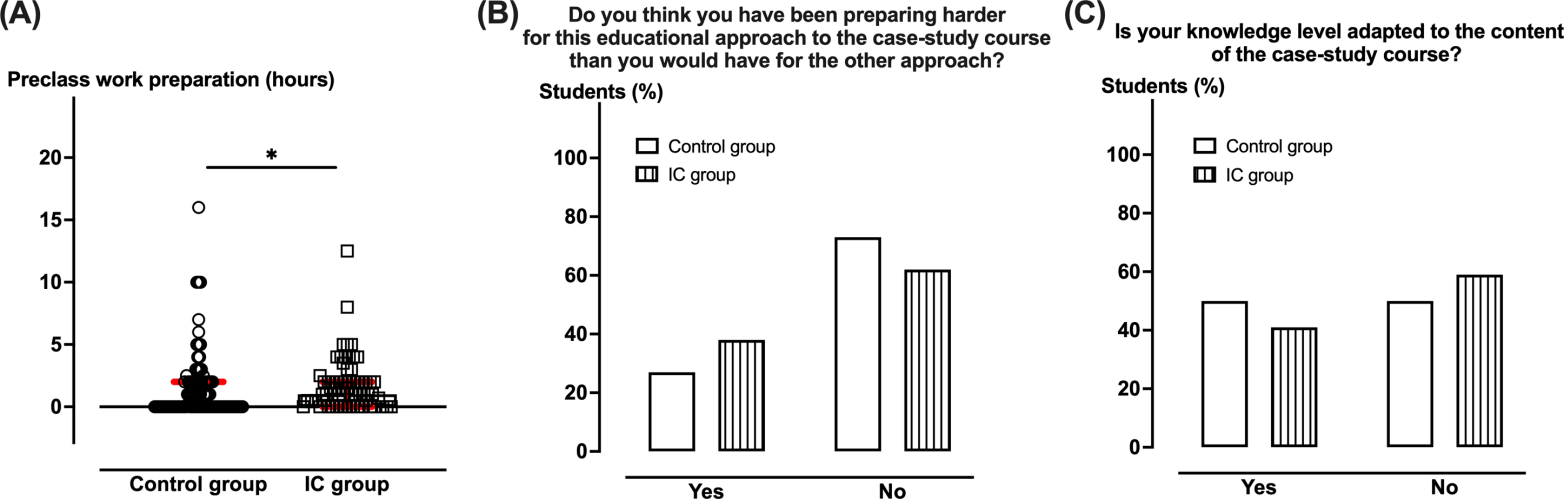
Self-assessment of the preclass work per group. (**A**) Preclass preparation requirements concerning working hours, (**B**) student perception, and (**C**) knowledge level self-assessment were compared between the control and IC groups. Red bars reflect median values with IQRs. While preclass work preparation necessitated more time for the IC compared to the control groups (*P*=.02), no difference was observed between the 2 groups in terms of work difficulty perception and the self-assessment of the knowledge level adapted to the course. IC: inverted classroom.

Students of both groups were asked to provide the number of hours spent preparing for the course, their perception of the preclass preparation difficulty in comparison to the other educational approach as well as the self-assessment of the required skill level. Preclass workload was estimated at 1 (IQR 0-2) hour in the IC group as a median, which was significantly higher (*P*=.02) than in the control group, 0 (IQR 0-2) hour (Figure 4A). While 38% of the students in the IC group considered that they had been preparing harder for the case-study course than it would have been if they were in the control group, 27% of the latter considered that as such it resulted in no significant difference between both groups (Figure 4B). Approximately half of the students in each group considered having a knowledge level adapted to the case-study course content. No difference was observed between both groups (Figure 4C).

### Empathy Self-Assessment

**Figure 5.**
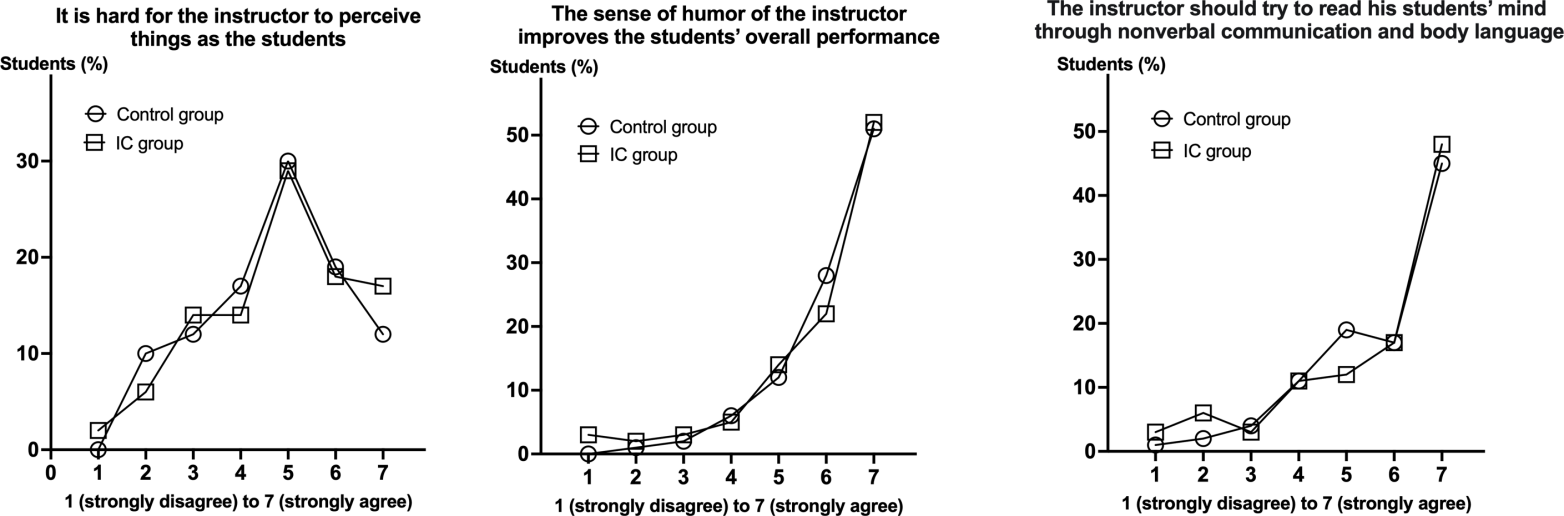
Empathy assessment questionnaire. The empathy of students for the instructor was assessed using 3 questions completed at the end of the case-study course. No difference was observed between the control and the IC groups for any of the 3 raised issues. IC: inverted classroom.

Student empathy for their instructor favors engagement and learning behavior in class, hence 3 related questions rated by a 7-point Likert scale were completed by students of both groups at the end of the course (Figure 5). Although only students in the IC group assumed the role of the instructor within the class, students’ opinions were quite similar between both groups. Approximately 30% of the students considered that it is hard for the instructor to perceive things as the students while 50% confirmed the importance of the sense of humor of the instructor in enhancing students’ performance and of reading the students’ minds through nonverbal communication and body language. These results did not differ between both groups.

### Global Satisfaction Assessment

**Figure 6.**
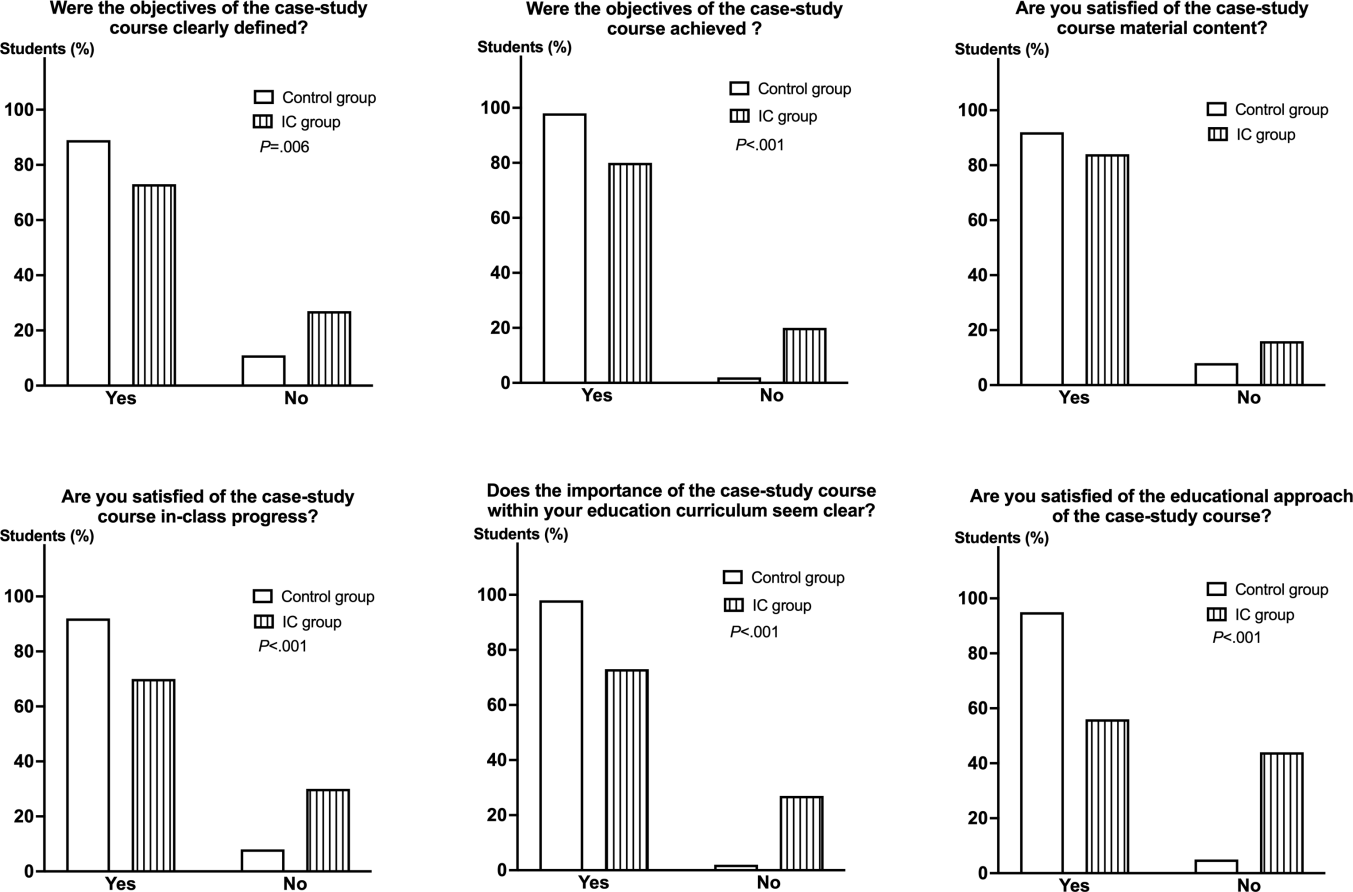
Satisfaction survey. A 6-item questionnaire relative to the students’ satisfaction with the pedagogical strategy (ie, traditional [n=93] vs IC [n=65]) was completed at the end of the case-study course. Overall, students in the control group were significantly more satisfied than those in the IC group. IC: inverted classroom.

The students’ global satisfaction was evaluated at the end of the course using a 6-item questionnaire (Figure 6). While more than 80% of the students were satisfied with the content of the course material in both groups, the satisfaction survey revealed that the IC approach was not well received among the students. Indeed, 18/65 (28%) of the students found that the objectives of the case-study course not clearly defined and 13/65 (20%) considered that they were not achieved, in comparison to 10/93 (11%*; P*=.006) and 2/93 (2%*; P*<.001) in the control group, respectively. A total of 20/65 (30%) of the students in the IC group were not satisfied with the in-class progress of the course versus 7/93 (8%) in the control group (*P*<.001). While 91/93 (98%) of the students in the control group considered this case-study course on antithrombotic drug use important for pharmacy education, only 47/65 (72%) did so in the IC group (*P*<.001). When it came to the question of the overall satisfaction of the educational approach, 88/93 (95%) of the students in the control group were satisfied versus (36/65) 55% in the IC group (*P*<.001).

## Discussion

### Principal Findings

Education methodology is increasingly shifting from a teacher- to student-centered learning approach [37]. In this context, we sought to assess whether applying an IC approach for a case-study course has an added value for conveying optimal antithrombotic drug use skills to pharmacy students. Such an educational approach would increase the communication, critical-thinking, problem-solving, and self-learning skills of pharmacy students. Our study revealed that while the IC approach did not increase student stress, it did not enhance their short-term knowledge retention or their empathy for the instructor. It increased the preclass workload and was not well received among the students. Of note, the case-study course on antithrombotic use was the first on this topic in the PharmD curriculum.

For any educational method to be considered successful, there must be evidence that student learning is enhanced. Many small-scale studies showed enhanced student learning following flipping lecture-based pharmacy courses [5-11,24]. Here, the IC approach applied to the case-study course was not associated with an enhanced preclass knowledge level and did not improve the students’ short-term knowledge retention. This was also the case in the Everly and Cochran study in which no significant differences in examination question performance between students in the lecture-based section and the flipped format section were observed [9]. That said, MCQ and examination scores are often used as a proxy measure for learning; however, the ultimate goal is for students to be able to apply classroom learning to real-life situations, which is more difficult to assess. Perhaps we need to examine other nonquantitative student characteristics outcomes following the IC approach in case-study courses (such as intellectual curiosity, personal responsibility, reasoning skills, etc) to identify pharmacy students most likely to succeed.

A recent systematic review including 45 studies with a total of 8426 students from various health professional pathways showed that implementing flipped classes may improve academic performance, and may support student satisfaction, yet the certainty of the evidence is low [37]. A second systematic review and meta-analysis including 11 randomized controlled Chinese studies enrolling 1200 participants suggested that flipped classroom pedagogy enhances students’ learning enthusiasm, self-learning ability, thinking and communication skills as well as cooperative ability [38]. Although, another systematic review focusing on students in pharmacy education, incorporated 6 observational studies with 1395 participants. No overall significant difference in final academic performance between the 2 educational models was reported [39]. An important heterogeneity of student perspectives from flipped classrooms has emerged in the literature, ranging from positive [10,24,40-42] to negative [21,39,43,44] and mixed [45] perceptions. These differences are potentially due to the different contexts in which these studies were carried out as well as different student populations, backgrounds, sample sizes, and outcome measures. Apart from that, some instructors may be more effective teachers than others regardless of the teaching modality. Research evaluating which elements contribute to the efficacy of an IC approach in pharmacy education is still needed. Moreover, it is still unclear if there is a particular area or topic that is better suited for the use of the IC approach in the PharmD curriculum. Besides, with the increased use of the IC method, it is important to consider the impact on students when this approach is incorporated into multiple concurrent courses. Determination of the ideal amount of preclass preparation time across the curriculum would provide helpful guidance to pharmacy faculties implementing such teaching methods.

One of the major limitations of the IC approach is its high dependence on the attendance to class time and on student engagement and discipline for reading and preparing the preclass material as previously emphasized [26]. As the class time attendance is not obligatory in our faculty, only 46% of the students were present in the antithrombotic drug use case-study course. The percentage of attendance was more important in the control group than in the IC group. A more elaborate strategy for students’ motivation should thus be implemented to obtain a higher engagement and adherence to our case-study courses in general, and to such IC experience if it shall be repeated. Providing clear expectations to students, keeping the preparation tasks focused, and explicitly linking preparation activities to in-class active learning could be some key methods for instructors to increase the proportion of students who prepare for classes. Future research should also be devoted to assessing the potential effect of sex, gender, socioeconomic background, and age on the outcomes of such an approach in the PharmD curriculum.

Preclass preparation could be considered as a considerable “extra” work [10,29,30,46]. Indeed, students in the IC group of our study reported an increased preclass workload which might take up an amount of their spare time leading to negative feedback on this approach. It is to be mentioned that the course materials made available beforehand should not be too complex to be understood by the students on their own. We did our best to include 6 uncomplicated cases issued from real-life settings and incorporate few slides to recall knowledge learned in the three lecture-based courses during the month preceding the case-study course.

Case-study courses with an IC approach are probably important in pharmacy education as they would help students promote higher-level critical-thinking skills, foster analyzing, and improve their communication skills therefore improving their motivation and attitudes [47]. Assisting them in learning how to think and communicate like a graduated pharmacist will prepare them for their future beyond pharmacy school. Communication skills are required to ensure patient understanding and compliance [48,49]. If a patient does not understand the purpose of the antithrombotic treatment, adherence will likely be low. Communication training in pharmacy students is thus mandatory to improve their later effectiveness as future health professionals. Although third-year students were overall not satisfied with this experience, such an approach is worth being retested with “older students,” from the fourth to the sixth year of the PharmD curriculum. Successful pharmacy students are expected to have the ability to manage their learning and adequately communicate their knowledge. Self-learning skills are particularly crucial to achieving effective lifelong learning in pharmacy, where scientific knowledge is continually evolving. Consequently, pharmacy students should be trained to be effective self-learners. Antithrombotic drug use assessed using a case-study course is an application-based activity, while the material taught previously is mostly a knowledge-based presentation. Therefore, third-year students may have significant difficulty in providing an application-based activity when their skill level may have been still on a much lower level of Bloom’s taxonomy [4] which may explain the low postclass knowledge scores in both groups. They will complete 2 additional learning years where it is anticipated they will gain more knowledge, clinical experience, and communication skills through their traineeships in community-based and hospital pharmacies as well as clinical laboratories. That said, many students were very interested in participating in this pedagogical study and found the experience to be innovative and enjoyable.

From the instructor’s perspective, the IC approach might be more challenging than a traditional session with a question-and-answer approach due to the risk that students’ presentation and case discussion activities create an unsettled classroom, thus a chaotic environment in which students may feel lost, and the fear that students may be unable to deliver the course adequately. However, the IC approach makes student engagement in the course easier and empowers them as active participants in their learning in comparison to the traditional educational approach. It allows the instructor to guide students in deeper learning processes as previously shown [38,46]. A more interactive teaching strategy may be more attractive than the IC approach, such as the adventure game recently developed by Perrin et al [50]. It is a video game in which the player assumes the role of a protagonist in an interactive story driven by exploration and problem-solving tests. Briefly, the pharmacy student assumes the role of a hematology superhero named SUPER HEMO. SUPER HEMO can meet 5 unwell characters in 5 different steps. The player must answer their questions and find the best way to diagnose and cure them. At the final hematology evaluation, students who played SUPER HEMO had a slightly better (but not statistically significant) median knowledge score than those who did not [50]. The value of such an innovative strategy for case-study courses on antithrombotic drug use in the PharmD curriculum remains to be established. Team-based learning is another educational approach that provides structure, defined timeframes, and formative assessment opportunities. It was previously shown to develop students learning enthusiasm, self-study, and thinking abilities as well as communication skills [51]. Therefore, it might be considered as an alternative approach for improving case-study courses in the PharmD curriculum, and thus is worth being assessed.

One possible limitation of our study is that the long-term effect of our IC approach on knowledge retention and skill application was not assessed. The question relative to this course was deliberately not included in the final exam in order not to create any lack of equity among the students of both groups. We did not complete any postclass knowledge survey 3 to 6 months later, nor did we assess the acquired skills through, for instance, an objective structured clinical examination. This remains to be specifically investigated. Second, we did not collect data on how many students effectively accessed the course material before the in-class session, although it might be feasible via the information technology service. As the preclass results of the students in the IC group were not better than those of the control group, we hypothesized that a lot of students had not read the course material before the target day. Noteworthily, it is hard to control whether students had effectively read and prepared the course material before the in-class session. Students might only click on the material folder without reading it or read parts of it. Also, several students might have accessed the material via 1 student user ID. We also did not ask students in the presurvey questionnaire whether they had read the assignment. However, they would, most probably, have not told the truth. Third, ideally, students in the IC group should have been asked to prepare the course material by themselves, yet this was not the case to avoid a high level of absenteeism as in-class attendance is not mandatory according to Paris Cité University policy. Despite this, a relatively small number of students effectively participated in this study. Fourth, in-class sessions with the traditional approach were completed 1 week before those with the IC approach to prevent students in the former group from having access to the material course from those of the latter group before the in-class session. Consequently, we cannot rule out the possibility that some students initially assigned to the IC group had changed their group assignment to get the case-study course at an early date. Finally, our findings cannot be generalized to other contexts, particularly to students in other year cohorts or to other specialties. This remains to be specifically investigated.

### Conclusions

Our study showed that an IC approach does not appear to be suited to the case-study course on antithrombotic drug use in the third-year PharmD curriculum. While no additional gain in short-term knowledge was observed using this approach in comparison to the traditional educational approach, we perceived significantly lower student satisfaction. However, the increased instructor-student and student-student interactions are still convincing arguments to try this pedagogical approach. Hence, additional research in this field is still needed to implement innovative educational approaches aiming at improving the knowledge and skills of our future pharmacists.

## Supplementary material

10.2196/67419 Multimedia Appendix 1 Pre- and postclass multiple-choice questions.

10.2196/67419 Multimedia Appendix 2 Stress self-assessment survey

10.2196/67419 Multimedia Appendix 3 Empathy assessment questionnaire

10.2196/67419 Multimedia Appendix 4 Preclass workload self-assessment and satisfaction questionnaire

10.2196/67419 Checklist 1 CONSORT 2010 checklist. CONSORT: Consolidated Standards of Reporting Trials.

## Abbreviations

IC: inverted classroom
MCQ: multiple-choice question
PharmD: Doctor of Pharmacy
