# Supplementary material for: Evaluation of the Inverted Classroom Approach in a Case-Study Course on Antithrombotic Drug Use in a PharmD Curriculum: French Monocentric Randomized Study
Source: JMIR Med Educ. 2025 Apr 10;11:e67419. doi: 10.2196/67419 (PMC12039941; doi:10.2196/67419)
Supplement: Multimedia Appendix 1 [file mededu-v11-e67419-s001.docx]

Comparison of inverted with traditional classroom approaches for a case-study course for third-year pharmacy students - ***protocole DPI-C***

**Pre- and post-class multiple-choice questions**

Last name: …………………………. First name: ………………………….

Course date: ……………………… Course schedule: …………………..

**Question 1**

Pre-therapeutic laboratory assays before starting anticoagulant therapy should include

- Complete blood count
- Prothrombin time, Factor V activity, anti-Xa activity
- Prothrombin time, activated partial thromboplastin time
- Plasma electrolytes (sodium, potassium, chloride)
- Serum creatinine (allowing creatinine clearance calculation)

**Question 2**

Following orthopedic surgery (knee or hip replacement surgery), the antithrombotic therapy

- is elective, depending on case-by-case assessment
- may be based on heparin therapy followed by vitamin K antagonist therapy
- may be based on antiplatelet therapy
- may be based on direct oral anticoagulant therapy
- is mandatory

**Question 3**

The laboratory monitoring of anticoagulant therapy

- based on unfractionated heparin at therapeutic dose is always mandatory
- based on vitamin K antagonist is performed using INR
- based on fondaparinux is mandatory
- based on direct oral anticoagulant might be necessary in very specific cases (e.g. severe bleeding)
- based on low molecular weight heparin enables dose adjustment in every patient

**Question 4**

Treatment of acute venous thromboembolic event

- may be based, from the onset, on direct oral factor Xa inhibitors
- may be based on low molecular weight heparin in case of a creatinine clearance below 15 mL/min
- may be based on apixaban 2.5 mg b.i.d. if the patient weight is below 60 kg
- be based on unfractionated heparin followed by vitamin K antagonist
- may be based on vitamin K antagonist with administration of a loading dose

**Question 5**

In case of non-valvular atrial fibrillation in an 81-year-old woman

- anticoagulant treatment is elective
- systemic embolism and stroke prevention is based on aspirin therapy
- direct oral anticoagulants may be prescribed as second-line drugs
- a vitamin K antagonist may be prescribed with no prior heparin therapy
- treatment with unfractionated heparin is commonly prescribed if the creatinine clearance is above 15 mL/min
