# Supplementary material for: Evaluation of the Inverted Classroom Approach in a Case-Study Course on Antithrombotic Drug Use in a PharmD Curriculum: French Monocentric Randomized Study
Source: JMIR Med Educ. 2025 Apr 10;11:e67419. doi: 10.2196/67419 (PMC12039941; doi:10.2196/67419)
Supplement: Multimedia Appendix 2 [file mededu-v11-e67419-s002.docx]

Comparison of inverted with traditional classroom approaches for a case-study course for third-year pharmacy students - ***protocole DPI-C***

**Stress self-assessment survey**

Course date: ………………………

**Question 1**

I had sleep disorders during the week preceeding the in-class session

- Very often
- Often
- Rarely
- Never

**Question 2**

I had a feeling of fear during the week preceding the in-class session

- Yes, seriously
- Yes, not that serious
- A bit, not so worrying
- Never

**Question 3**

I was nervous during the week preceding the in-class session

- Very often
- Often
- Rarely
- Never

**Question 4**

On the D-Day, I cared about my appearance

- More than usual
- As usual
- Not at all

**Question 5**

I had a sudden feeling of panic during the week preceding the in-class session

- Very often
- Often
- Rarely
- Never

**Question 6**

I was annoyed that things turned out-of-control during the week preceding the in-class session

- Very often
- Often
- Rarely
- Never
