# Supplementary material for: Evaluation of the Inverted Classroom Approach in a Case-Study Course on Antithrombotic Drug Use in a PharmD Curriculum: French Monocentric Randomized Study
Source: JMIR Med Educ. 2025 Apr 10;11:e67419. doi: 10.2196/67419 (PMC12039941; doi:10.2196/67419)
Supplement: Multimedia Appendix 3 [file mededu-v11-e67419-s003.docx]

Comparison of inverted with traditional classroom approaches for a case-study course for third-year pharmacy students - ***protocole DPI-C***

**Empathy assessment questionnaire**

Course date: ………………………

Rate the following items by a 7-point Likert scale (1 = strongly disagree, 2 = disagree, 3 = somewhat disagree, 4 = neutral, 5 = somewhat agree, 6 = agree, 7 = strongly agree)

**Question 1**

It is hard for the instructor to perceive things as the students

……

**Question 2**

The sense of humor of the instructor improves the students’ overall performance

……

**Question 3**

The instructor should try to read his students mind through non-verbal communication and body language

……
