# Supplementary material for: Evaluation of the Inverted Classroom Approach in a Case-Study Course on Antithrombotic Drug Use in a PharmD Curriculum: French Monocentric Randomized Study
Source: JMIR Med Educ. 2025 Apr 10;11:e67419. doi: 10.2196/67419 (PMC12039941; doi:10.2196/67419)
Supplement: Multimedia Appendix 4 [file mededu-v11-e67419-s004.docx]

Comparison of inverted with traditional classroom approaches for a case-study course for third-year pharmacy students - ***protocole DPI-C***

**Pre-class workload self-assessment and satisfaction questionnaire**

Course date: ………………………

**Question 1**

Were the objectives of the case-study course clearly defined?

- Yes
- No

**Question 2**

Were the objectives of the case-study course achieved?

- Yes
- No

**Question 3**

Does the importance of the case-study course within your education curriculum seem clear?

- Yes
- No

**Question 4**

Is your knowledge level adapted to the content of the case-study course

- Yes
- No

**Question 5**

Are you satisfied of the educational approach of the case-study course?

- Yes
- No

**Question 6**

Are you satisfied of the case-study course in-class progress?

- Yes
- No

**Question 7**

Are you satisfied of the case-study course materiel content?

- Yes
- No

**Question 8**

How many hours of pre-class preparation were necessary for this case-study course?

………………………

**Question 9**

Do you think you have been preparing harder for this educational approach of case-study course than it would have been for the other approach?

- Yes
- No
